# Choosing the Best Plant for the Job: A Cost-Effective Assay to Prescreen Ancient Plant Remains Destined for Shotgun Sequencing

**DOI:** 10.1371/journal.pone.0045644

**Published:** 2012-09-20

**Authors:** Nathan Wales, J. Alberto Romero-Navarro, Enrico Cappellini, M. Thomas P Gilbert

**Affiliations:** 1 Department of Anthropology, University of Connecticut, Storrs, Connecticut, United States of America; 2 Centre for GeoGenetics, Natural History Museum of Denmark, Copenhagen, Denmark; 3 Undergraduate Program on Genomic Sciences, Universidad Nacional Autónoma de México, Cuernavaca, Mexico; Institut de Biologia Evolutiva - Universitat Pompeu Fabra, Spain

## Abstract

DNA extracted from ancient plant remains almost always contains a mixture of endogenous (that is, derived from the plant) and exogenous (derived from other sources) DNA. The exogenous ‘contaminant’ DNA, chiefly derived from microorganisms, presents significant problems for shotgun sequencing. In some samples, more than 90% of the recovered sequences are exogenous, providing limited data relevant to the sample. However, other samples have far less contamination and subsequently yield much more useful data via shotgun sequencing. Given the investment required for high-throughput sequencing, whenever multiple samples are available, it is most economical to sequence the least contaminated sample. We present an assay based on quantitative real-time PCR which estimates the relative amounts of fungal and bacterial DNA in a sample in comparison to the endogenous plant DNA. Given a collection of contextually-similar ancient plant samples, this low cost assay aids in selecting the best sample for shotgun sequencing.

## Introduction

The field of ancient DNA (aDNA) has provided unparalleled insights into many anthropological, archaeological, and paleontological questions, including evolution, domestication, and demography [1]–[3]. While great strides have been made in understanding DNA preservation and degradation [4]–[6], one issue that continues to hinder aDNA research is contamination [7]–[9]. Unlike modern DNA samples, ancient specimens are characterized by low DNA concentrations and highly fragmented DNA molecules [10], [11]. Consequently, the small amount of endogenous DNA in a sample can be easily overwhelmed by ubiquitous modern DNA. For this article, we employ a broad definition of contamination, extending it to include all DNA derived from sources other than the expected organism. In this way, contaminant DNA may originate from modern sources, such as personnel and laboratory reagents, but also from organisms which consumed sample tissues post-mortem and soil organisms that infiltrated macroremains or covered their surfaces. This definition is useful because DNA derived from sources other than the species of interest generally provides little useful information for evolutionary questions. Ancient DNA researchers must assume that almost all samples are contaminated to some extent; however, the consequences of that contamination depend on many factors, including: the species of interest, the depositional context, curation of the specimen, and the experimental methodology.

Over the past two decades, the majority of aDNA research has relied upon PCR-based experiments to study small numbers of loci of interest [12]. This approach limits the effects of most contaminants because target-specific primers selectively isolate and amplify a particular gene or marker in the genome of interest. Extensive contamination is thereby overwhelmed, allowing PCR amplicons to be readily used in downstream applications like bacterial cloning and Sanger (dideoxynucleotide) sequencing [13].

In 2005, the direction of DNA sequencing was changed with the introduction of the Roche/454 FLX high-throughput sequencing platform [14]. Using this technology, Poinar *et al.* [15] recovered 13 million base pairs (bp) of endogenous DNA from 40,000 year-old woolly mammoth (*Mammuthus primigenius*) fossils. Later platforms like the Illumina GA/HiSeq series and Life Technologies SOLiD series continued the trend and have infiltrated all forms of DNA research because of their flexibility, cost-effectiveness, and ground-breaking data production [16]. High-throughput DNA sequencing has been invaluable for many aDNA research projects, notably leading to the genomes of the woolly mammoth, Neanderthal, Denisova hominin, a Greenland Paleo-Eskimo man, and an Australian aborigine [3], [17]–[20]. The so-called “sequencing revolution” [21] has been a boon to recovering paleogenomes, but it also forces researchers to reconsider the impacts of contamination in ancient samples.

The most straightforward use of high-throughput sequencing on ancient samples is shotgun sequencing [12]. More complicated approaches, such as targeted capture, have become important for some high-profile aDNA projects, including the Neanderthal genome [22], [23]. However, such experiments tend to be technically challenging and require lengthy optimization. In contrast, shotgun sequencing can be implemented relatively easily in most aDNA laboratories. For plant aDNA research, this approach can provide crucial information about domestication and plant evolution, as demonstrated by Palmer *et al.*’s [24] analysis of ancient cotton (*Gossypium* spp.). Shotgun sequencing of the cotton samples revealed species affiliation as well as insights into punctuated evolution via frequencies of transposable elements. When paired with a comprehensive reference database, shotgun sequencing can also provide enough information to allow missing data to be imputed, as is currently possible with human genomes [25]. High quality databases are becoming available for modern plants, such as maize (*Zea mays*) landraces [26], [27], and will likely become fundamental for aDNA research.

Since shotgun sequencing is in essence a random subsampling of the DNA molecules extracted from a specimen, it reflects the abundance of DNA from exogenous sources. In the first publication of high-throughput experiments on ancient plant remains, Ávila-Arcos *et al.* [28] found large disparities in endogenous DNA content between samples: 25% and 11% endogenous DNA in two 1,400-year-old maize cobs, but >90% in 700-year-old maize kernels. Shotgun sequencing by Palmer *et al.* [24] recovered 95% and 64% endogenous DNA in 3,750 and 1,600-year-old cotton seeds. In contrast, <4% of the sequences from a 1000-year-old cotton seed matched the expected genome; however, extensive DNA damage likely prevented genus-level identification of many of the sequencing reads, and a value closer to 50% is more realistic. At the other end of the spectrum, Bunning *et al.’s* [29] high-throughput sequencing of a mixture of 3,000-year-old charred grains recovered <1% endogenous DNA. This considerable variability in endogenous DNA content in ancient plant samples is important because it determines the effectiveness of shotgun sequencing, as exogenous DNA is essentially useless. At present, researchers must arbitrarily sequence samples of unknown quality, and hope the resulting data is sufficient to answer their research questions. When sub-optimal samples are sequenced, additional sequencing runs may be necessary to reach statistically significant thresholds, an expensive and time-consuming proposition.

Therefore, it is advantageous to have an indication of the levels of contamination in a collection of plant specimens that might be under consideration for shotgun sequencing. If several samples originate from similar contexts and are expected to provide equivalent scientific insights via shotgun sequencing, there is an obvious benefit for choosing the least contaminated specimen. In order to determine the best candidate for shotgun sequencing, we have developed and tested an assay to estimate the relative levels of contamination in ancient plant samples, such as archaeobotanical remains or herbarium specimens. The assay is based on the sensitivity of real-time quantitative PCR (qPCR) to determine the relative amount of bacterial, fungal, plant chloroplast, and plant nuclear DNA in a sample. Both chloroplast and nuclear plant DNA are measured because the number of chloroplasts in a cell depends on the tissue type [30] and different research goals may emphasize one genome over the other.

## Materials and Methods

### DNA Extraction and Illumina Library Preparation

Samples were prepared in a dedicated aDNA clean laboratory at the University of Copenhagen, following stringent conventions required by the discipline [11]. DNA was isolated in an organic extraction using the following protocol [31]:

1. Washed 1 seed or ∼100 mg non-porous plant remains in 0.5% bleach (NaClO) for 30 seconds, followed by a rinse in molecular-grade water. Porous samples, such as maize cobs, were cleaned by removing the external surface with a sterile scalpel.
2. Crushed or diced plant remains using sterile implements.
3. Digested plant remains overnight at 55°C in 750 µL buffer consisting of 10 mM Tris-HCl, 10 mM NaCl, 2% w/v SDS, 5 mM CaCl_2_, 2.5 mM EDTA, 40 mM DTT, and 10% Proteinase K.
4. Extracted DNA using two rounds of phenol and one round of chloroform.
5. Cleaned and concentrated DNA using MinElute PCR purification kit (Qiagen, Valencia, CA).

Following extraction, DNA was converted into Illumina GAII-compatible libraries using the designated NEBNext library building kits for second generation sequencing (New England Biolabs, Ipswich, MA; catalogue number: E6040S, E6090S). Libraries were prepared and amplified according to manufacturer’s directions, with 18–25 PCR cycles.

Although non-amplified libraries or unmodified DNA extracts can be tested in the assay, we focused experimentation on amplified libraries for several reasons. First, in order to determine the level of contamination in a sample, an amplified library is required for shotgun sequencing on the most common second generation high-throughput platforms. This presents an obstacle because amplification biases, such as differential primer affinity or PCR drift, can lead to different relative frequencies of molecules in amplified libraries versus the original template [32], [33]. By using the exact same solution in the assay and shotgun sequencing, the consequences of amplification biases are avoided. Second, using amplified libraries in the contamination assay also reduces the likelihood that enzymatic inhibitors co-extracted with DNA will interfere with the qPCR experiment because such inhibitors are further diluted or removed in the process of library construction. This precaution is particularly important because many ancient plant samples will not amplify in PCR without the additive bovine serum albumin (BSA); however, BSA may interfere with the detection of fluorescence by the qPCR camera. Nonetheless, some ancient plant DNA extracts were tested in the assay with BSA and were found to function properly, as discussed below.

### Real-time qPCR Assay

The contamination estimation assay was developed and tested on a Roche LightCycler 480 Real-time PCR System using SYBR Green chemistry. This qPCR approach was selected because it is less expensive and more flexible than fluorescence probes, such as TaqMan. SYBR Green molecules fluoresce when bound to double-stranded DNA, and therefore can be used in any number of laboratory assays simply by changing primer sets, thereby allowing small scale testing of different qPCR experiments without needing to maintain a stock of various probes at all times. Note that SYBR Green dye also fluoresces in the presence of primer-dimers, which may form even in the absence of PCR products, so this phenomenon must be taken into account when interpreting qPCR results.

Four sets of oligonucleotide primers were used to target markers in bacterial, fungal, plant chloroplast (cpDNA), and plant nuclear DNA (nuDNA), as listed in Table 1. Other sources of contamination, such as common laboratory mammals and human DNA, generally comprise a very small percentage of DNA sequences found in ancient plant samples (often <1%), and therefore are not measured in the contamination assay. However, it must be recognized that Bunning *et al.’s* [29] shotgun sequencing of a mixture of ancient charred cereals found 67% of the identifiable DNA to be derived from animals, predominantly humans and mice. As charring fragments and damages DNA [34], minute quantities of endogenous DNA can be easily overwhelmed by contaminants; therefore, genetic testing of charred samples should be conducted with caution, although the assay might still aid in selecting between charred samples.

**Table 1:** Primers for qPCR contamination assay.

Targeted genome or organism	Primer sequence	Length (bp)	Ref.
Plant nuclear (tRNA-His gene)	F: TGTGGCTGCTGGGATTCGAGC	50	This study
	R: AATTCCACGTTGTGGCCGTGGA		
Plant chloroplast (*rbcL* gene)	F: GGCAGCATTCCGAGTAACTCCTC	138–140	[35]
	R: CGTCCTTTGTAACGATCAAG		
Bacteria (16S rRNA gene)	F: GGAGTACGGCCGCAAGGT	65	[36]
	R: CATGCTCCACCGCTTGTG		
Fungi (18S rRNA gene)	F: AGATACCGTCGTAGTCTTAACCATAAACT	131–132	[37]
	R: TTCAGCCTTGCGACCATACT		

The primer set for plant nuDNA was designed for this assay and amplifies the gene coding for the Histidine tRNA molecule. This short gene is highly conserved due to its important function in DNA translation and is compatible with the short length of aDNA. Primers designed for the 72 bp tRNA-His gene in thale cress (*Arabidopsis thaliana*) (NCBI Gene 3771556) were found, *in silico*, to be compatible with known sequences of flowering plant species as diverse as tomato (*Solanum lycopersicum*), rice (*Oryza sativa*), and grapes (*Vitis vinifera*). Importantly, non-plant species do not have regions of their genomes that will amplify with the primer set. Other conserved nuclear loci which might serve as universal primer binding sites, such as genes for other tRNA molecules, histones, RNA polymerases, elongation factor 1-alpha, and alcohol dehydrogenase, were also tested, but few showed the promise of tRNA-His.

The cpDNA primers, designed by Poinar *et al.* [35], amplify a fragment of the chloroplast ribulose-bisphosphate carboxylase (*rbcL*) gene. These primers perfectly match the primer-binding sites in most angiosperms, and have only 1 bp difference in most other flowering plants as well as some conifers. Importantly, green algae have at least 4 bp differences with one primer, according to the NCBI-nt nucleotide database. Due to partial binding of primers to the *rbcL* gene in algae, the marker may potentially amplify, but with less efficiency than in terrestrial plants. Thus, if the primer set is used on waterlogged plant materials, it should preferentially amplify endogenous cpDNA instead of contaminant algae. It should also be noted that the cpDNA marker is more properly termed a plastome marker, as all plastids in a plant share the same genome. Therefore, the primers also work on plant tissues like roots, seeds, and branches because they contain leucoplasts, non-pigmented organelles involved in storage of starches, lipids, and proteins.

The bacterial and fungal primers are published by Oskam et al. [36] and Bell et al. [37], respectively. The bacterial primers amplify a portion of the 16S ribosomal RNA gene, a region known to be conserved among many bacteria. This primer set was originally developed to identify bacterial contamination in fossil egg shells and can detect both ancient and modern bacteria due to the short length of the targeted locus. Similarly, the fungal primer set targets a highly conserved region of the 18S rRNA gene, and is short enough to act as a generic marker for modern and ancient fungi.

**Table 2:** Archaeobotanical samples tested in assay.

Name	Species	Tissue	Context	Library PCR details
AR 6	*Vitis vinifera*	Desiccated branch	Areni I cave, Armenia. Medieval context.	22 cycles
FE 2599	*Vitis vinifera*	Desiccated seed	Porta Remo-Via Vespergolo site, Ferrara, Italy.Stratigraphic unit 2599, dated by artifacts tofirst half 11th century AD.	22 cycles
AZ 935	*Zea mays*	Desiccated kernel	Turkey House Ruin, Arizona. 707±23 ^14^CYBP. [28]	20 cycles
PLM 4	*Zea mays*	Desiccated kernel	Playa Miller 4 site, Chile. Dated to 750-550 YearsBP. [28]	22 cycles
MEX 1	*Zea mays*	Desiccated cob	Mexican archaeological site, unknownprovenance and unknown age.	22 cycles
CMAG 10189	*Zea mays*	Desiccated cob	Cueva del Maguey 1 site, Pueblo Nuevo, Durango,Mexico. Dated to 1410±25 ^14^CYBP. [28]	18 cycles
CMAG 10237	*Zea mays*	Desiccated cob	Cueva del Maguey 1 site, Pueblo Nuevo, Durango,Mexico. Dated to 1410±25 ^14^CYBP. [28]	25 cycles

Each 25 µL reaction contained 1 U AmpliTaq Gold polymerase (Applied Biosystems, Foster City, CA), 1X AmpliTaq Gold buffer, 2.5 mM MgCl_2_, 0.2 mM dNTPs, 0.4 µM primers, 1 µL 1X SYBR Green/ROX mix (Invitrogen, Carlsbad, CA), and 1 µL of template DNA. Cycling conditions for the qPCR assay were as follows: 95.0°C for 10 min enzyme activation, 50 cycles of 95.0°C for 30 s, 54.0°C for 1 min, and 72.0°C for 1 min, followed by a melting curve. In order to test expected amplification dynamics, each sample was tested in a dilution series, with template DNA at concentrations of 100%, 10%, and 1% (i.e., 1 µL of DNA eluate, 0.1 µL, and 0.01 µL). As discussed below, the dilution series can be used to identify inhibition and other experimental errors that may not be observed when only testing an undiluted library. Spreadsheet S1 can be used to prepare the assay, including calculations for master mix setups and recommended microwell plate layout.

qPCR was performed on the Roche LightCycler using default settings to observe when the fluorescence of a given marker exceeds the background fluorescence. The cycle threshold (C_t_) values were determined by the LightCycler software using the second derivative maximum method and high sensitivity algorithm. Rather than computing an absolute number of template molecules for the bacteria, fungi, chloroplast, and plant nuclear markers, the relative levels were determined using differences in C_t_ values. This decision was made as ultimately absolute copy number is a factor dependent on the quantity of material extracted, and in most situations is less important for shotgun sequencing of ancient samples than endogenous DNA content. Assuming perfect amplification efficiency, each PCR cycle doubles the copy number of the marker of interest. Thus, for example, if the bacteria and chloroplast markers in a sample have C_t_ values of 21 and 24, respectively, the sample started with eight times more copies of the bacterial locus than chloroplast locus. In an ideal situation where the genome sizes of bacteria and chloroplast were equal, correspondingly there would be eight times more bacterial DNA than chloroplast DNA. In reality, these genomes differ in size, preventing an exact prediction of the absolute difference in DNA quantity between the bacteria and chloroplast. With regards to the utility of this assay, however, as long as the genomes of the different targets are relatively similar between different samples under study, the difference in C_t_ values can still be used to compare contamination levels.

For the purpose of this assay, in order to derive a simple means of ranking/comparing samples, despite the above caveat, we assume the simplistic situation where genome sizes of contaminant and endogenous DNA are equal. Thus, the first marker to cross the threshold was identified as the most common component and other markers were calculated as a ‘percentage’ of the maximum using Equation 1, with the assumption of perfect amplification efficiency:
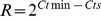
(1)where R is the relative amount of DNA, C_t min_ is the minimum crossing point value, and C_t s_ is the crossing point value of a given marker. In the above example, the relative amount of the chloroplast DNA marker amplified is 12.5% compared to bacterial DNA marker amplified [2^21–24^ = 2^−3^ = 1/(2^3^) = 0.125]. However, it is important to remember, that due to both the discussion outlined above, plus inefficiencies and related issues in real world experiments, as discussed below, these percentages should not be assumed to be perfectly accurate, but rather approximate guides.

It is essential to determine whether a marker amplifies before the formation of primer-dimers in the negative control. Spreadsheet S1 contains an automated quality check of exported qPCR data and will identify unreliable readings from a sample. Alternatively, one may manually verify data by 1) observing whether amplification curves in samples rise about the background fluorescence before the corresponding negative control, and 2) checking if each 10% dilution crosses the fluorescence threshold after the higher concentration (3.32 cycles in a perfectly efficient reaction). Due to the presence of various sources of contaminant DNA, non-specific amplification may occur, resulting in the lower C_t_ values. Non-specific amplification can be identified by comparing melting curves of a given primer set for all tested samples; aberrant melting curves may indicate non-specific amplification of longer or shorter loci and should be omitted from analyses.

## Experiments and Results

### Verification of Assay

#### Assay of ancient plant samples

Seven ancient desiccated plant samples were tested in the assay to investigate its accuracy in quantifying contamination by bacteria and fungi. Archaeobotanical remains of grape (Vitis vinifera) and maize (Zea mays) samples were tested, ranging in age from 700 to 1400 ^14^C years before present. Detailed specimen and contextual information are found in Table 2. All specimens were tested in the assay in the manner described above. The relative amounts of DNA from different sources were calculated using Spreadsheet S1. The results of the assay are found in Table 3.

**Table 3:** Verification of assay on amplified libraries with shotgun sequencing data.

	qPCR assay results		Shotgun sequencing results		
Sample	Primer set	Relative to maximum^1^	Endogenous DNA	Mapping and BLASTfindings	Relative to maximum^2^
AR 6	Plant genome	2.26%	5.04%	*Vitis v.* nuDNA	13.85%
	Chloroplast	0.58%		*Vitis v.* cpDNA	0.66%
	Bacteria	Maximum		Bacteria	Maximum
	Fungi	0.75%		Fungi	3.06%
Fe 2599	Plant genome	*N/A* 3	0.14%	*Vitis v.* nuDNA	0.56%
	Chloroplast	11.34%		*Vitis v.* cpDNA	0.01%
	Bacteria	Maximum		Bacteria	Maximum
	Fungi	11.42%		Fungi	2.66%
AZ 935	Plant genome	Maximum	92.38%	*Zea m.* nuDNA	Maximum
	Chloroplast	25.53%		*Zea m.* cpDNA.	0.30%
	Bacteria	20.73%		Bacteria	0.32%
	Fungi	0.91%		Fungi	0.06%
PLM 4	Plant genome	Maximum	90.59%	*Zea m.* nuDNA	Maximum
	Chloroplast	7.75%		*Zea m.* cpDNA.	0.19%
	Bacteria	32.99%		Bacteria	0.55%
	Fungi	7.75%		Fungi	0.41%
MEX 1	Plant genome	*N/A*	80.86%	*Zea m.* nuDNA	Maximum
	Chloroplast	Maximum		*Zea m.* cpDNA.	0.15%
	Bacteria	11.10%		Bacteria	3.89%
	Fungi	0.31%		Fungi	0.46%
CMAG 10189	Plant genome	*N/A*	11.00%	*Zea m.* nuDNA	51.73%
	Chloroplast	*N/A*		*Zea m.* cpDNA.	0.08%
	Bacteria	Maximum		Bacteria	Maximum
	Fungi	41.18%		Fungi	18.49%
CMAG 10237	Plant genome	*N/A*	24.69%	*Zea m.* nuDNA	99.39%
	Chloroplast	2.52%		*Zea m.* cpDNA.	0.05%
	Bacteria	Maximum		Bacteria	Maximum
	Fungi	1.63%		Fungi	6.98%

1 As discussed in the methods section, the assay percentages are meant as a guide to compare samples and are not expected to match the absolute values yielded via shotgun sequencing.

2 The scaled shotgun sequencing results do not include reads without BLAST matches or reads which matched higher taxonomic levels (e.g., eukaryotes or metazoa).

3 *N/A* indicates the primer set did not fluoresce before the negative control for the sample.

#### Sequencing of ancient plant samples

The seven archaeobotanical samples tested in the assay were shotgun sequenced on individual lanes of an Illumina GAIIx sequencing platform. Sequencing reads were quality checked and clonal sequences were collapsed, as described in Ávila-Arcos et al. [28]. After data cleaning, samples yielded an average of 23.6 million reads (range: 12.9 M–38.6 M).

Shotgun sequencing reads were mapped against the chloroplast (grape: NCBI accession NC_007957; maize: NC_001666.2) and nuclear genome of the respective species (Vitis GenBank assembly: GCA_000003745.2; maize GenBank assembly: GCA_000005005.2) using the BWA bioinformatics package [38]. The percentage of sequencing reads which mapped to the nuclear genome was highly variable between samples, ranging from 0.37% to 92.11%. For all samples, less than 0.3% of reads mapped to the reference chloroplast. However, the number of chloroplast reads compared to nuclear DNA reads varied between 4.78% (AR 6 grapevine) to 0.05% (CMAG 10237 maize cob). Among other things, these numbers reflect differences in the relative sizes of the nuclear and plastid genomes. For example, the maize nuclear genome is 2,048 MB while the maize plastome is 140,387 bp, representing less than 0.007% of the length of the nuclear genome [39], [40]. However, plants have many plastids per cell, which leads to large variations in the ratio of nuDNA and cpDNA. Leaf cells contain ∼100 chloroplasts per cell on average, but the total number of plastids for a cell may vary from less than 50 to more than 500 depending on the particular species, tissue type, and developmental stage of a plant [41]–[43]. Furthermore, each plastid may have hundreds of copies of the plastome, ultimately ranging from ∼1000 copies of the plastome in leaf cells of thale cress to more than 10,000 copies in tobacco (*Nicotiana tabacum*) [44]. A mitigating factor can come from endopolyploidization, the departure from the normal ploidy level in mature cells caused by DNA replication without mitosis. For instance, cabbage (*Brassica oleracea*) flowers and thale cress leaves been recorded as having up to 32 and 128 copies of the nuclear genome in mature cells, respectively [45], [46]. Considering the differences in genome sizes and the high variability in the number of nuclear and plasmid genomes in plant cells, the shotgun sequencing data are generally consistent with the potential ratios of cpDNA to nuDNA. A random sample (n = 100,000) of the non-mapped reads from each specimen was compared against a local copy of NCBI-nt nucleotide database to determine their origins. The BLAST results were imported into MEGAN 4.66.4 [47] to explore the relative abundance of different organisms. Except for lowering the minimum bit score to 35, the default LCA parameters were used. The percentages of bacteria and fungi reads were extrapolated to the entire shotgun sequencing data, based upon the findings in MEGAN and the number of reads which did not map to the chloroplast or nuclear genomes. With these calculations, the most common type of DNA (plant nuDNA, cpDNA, bacterial, or fungal) in a sample was scored as 100% and the others were scored as a percentage of the maximum, as listed in Table 3. It should be noted that reads without a BLAST match and reads which matched higher taxonomic groups, such as eukaryotes, are not represented in the table.

### Application of Assay to Ancient DNA Extracts

While assay experimentation and verification was primarily focused on amplified DNA libraries, the qPCR assay was further tested with unmodified DNA extracts of three ancient plant samples: AR 6, AZ 935, and CMAG 10189. These extractions were conducted at a later date than the DNA libraries tested above. The exact same grape branch and maize cob were used for AR 6 and CMAG 10189; however, a different maize kernel was processed for AZ 935. As such, these samples may further depart from the corresponding shotgun sequencing data and assay results on the amplified libraries. The qPCR assay was conducted as done previously, except for the addition of 20 mg of molecular biology-grade BSA in each reaction to prevent enzymatic inhibition. The results of the assay are in Table 4.

**Table 4:** Verification of assay on DNA extracts.

	qPCR assay results	
Sample	Primer set	Relative to maximum
AR 6	Plant genome	1.24%
	Chloroplast	5.18%
	Bacteria	Maximum
	Fungi	75.79%
AZ 935	Plant genome	Maximum
	Chloroplast	8.30%
	Bacteria	3.56%
	Fungi	1.20%
CMAG 10189	Plant genome	*N/A*
	Chloroplast	0.01%
	Bacteria	5.63%
	Fungi	Maximum

## Discussion

The qPCR assay of seven ancient plant samples demonstrates a clear correspondence with shotgun sequencing data, especially for the criteria of endogenous DNA content. The two specimens with the highest endogenous DNA content, AZ 935 and PLM 4, are found to be the best samples in assay. For each of them, the plant genome marker has the lowest C_t_ value, and is identified as the maximum DNA contributor. Furthermore, the assay suggests the AZ 935 sample has lower levels of bacteria and fungi, which is consistent with the shotgun data. The third sample in terms of endogenous DNA content, MEX 1, is also identified as having high levels of cpDNA, although the nuclear primers failed to fluoresce before the negative control. Due to the formation of primer-dimers, this phenomenon cannot be avoided in every sample, but it can likely be ignored when chloroplast markers indicate high endogenous DNA content. The other samples with much lower endogenous DNA (<25%) are correctly identified in the assay as having most DNA derived from bacteria. While there are some trends for these low quality samples in relative levels of nuDNA and cpDNA, we are hesitant to read too much into the dataset. Rather, it should be assumed that due to the formation of primer-dimers, different amplification efficiencies, and related issues of PCR kinetics, the exact ratio of different DNA types (nuDNA, cpDNA, bacterial, and fungal) does not perfectly reflect those yielded by shotgun sequencing. Nonetheless, the best and worst candidates for shotgun sequencing can be readily determined by examining the results of the assay. It should be reiterated that although a sample may be identified as being “worse” than others, it does not mean the sample must be forever abandoned. For example, AR 6 is correctly identified having low amounts of endogenous DNA (5.04% endogenous DNA according to shotgun sequencing). If research goals change, it may be worthwhile to eventually sequence a sample like AR 6, and the assay can be used to predict how much useful data shotgun sequencing will yield.

The results for the three DNA extracts tested in the assay are largely consistent with those of the amplified libraries. For example, AZ 935 is again found to have the maximum contribution of DNA from nuclear plant DNA, and AR 6 and CMAG 10189 are identified as being mostly composed of exogenous DNA. Interestingly, CMAG 10189 is found to have more fungal DNA than bacterial DNA, the reverse of what is seen in the amplified library, a trend mirrored in AR 6 where fungi are more common in the extract than in the amplified library. There are a few possible explanations for these differences. First, the DNA libraries were constructed from earlier extractions in which the external portion of the branch and cob were freshly removed; the later experiments may have extracted fungi which colonized these areas in the intervening months. Second, if the contamination is by modern fungi, it is possible that their genetic material did not get incorporated into the amplified libraries because no DNA fragmentation steps were undertaken prior to library building, and PCR could have favored small endogenous molecules, ultimately swamping out the fungal signature. In contrast, AZ 935 is more consistent in fungal levels, perhaps because the exterior of the maize kernel was washed with bleach, a step with is not possible with desiccated branches and cobs. Regardless, the overall picture remains the same, and the sample with the greatest potential in the group, AZ 935, is readily identified. The second best sample, AR 6, is selected over CMAG 10189 due to the relatively higher levels of nuDNA and cpDNA. Even though it is possible to test DNA extracts in the assay, it is still preferable to assay amplified libraries. Results from aDNA extracts could be misleading because endogenous DNA and modern contaminants may not become incorporated into DNA libraries at the same rate and/or amplify at different efficiencies due to damage patterns or differences in length. The most reliable predictor of shotgun sequencing results will therefore come from tests on amplified libraries rather than aDNA extracts. While the construction and amplification of multiple libraries adds an additional expense, the resulting shotgun sequencing data will yield more endogenous DNA data and likely save resources in the long term.

**Figure 1:**
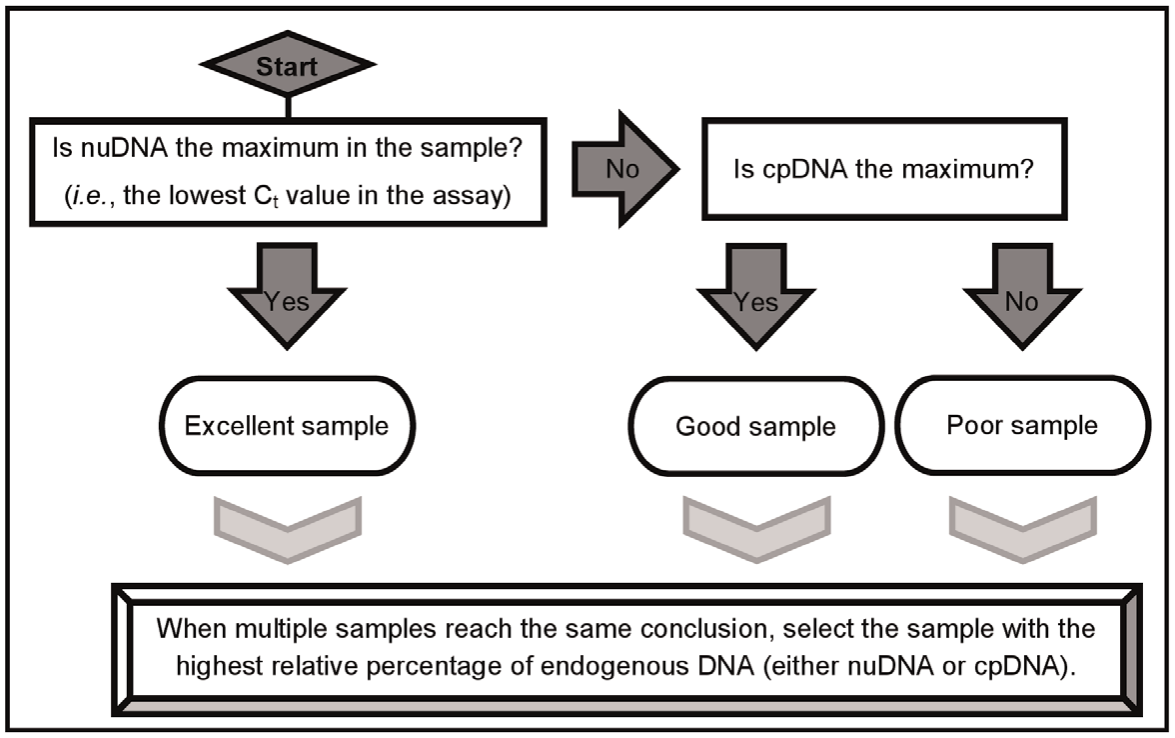
Directions to interpret qPCR assay data. Follow the questions in the flowchart to compare the endogenous DNA content of different ancient plant samples. The least contaminated samples are best suited for shotgun sequencing.

Figure 1 provides a simple way to compare the endogenous DNA content of different samples. This flowchart highlights the key findings of an experiment, and helps identify the best ancient plant samples for further analyses. It is critical to first ensure that C_t_ values are reliable before comparing samples, especially if Spreadsheet S1 is not used. If a C_t_ value for a sample is equal to the negative control for a given primer set, it is not valid and must be ignored. Likewise, C_t_ values should increase along a dilution series, although they may not exhibit ideal amplification efficiency. Deviations from these expectations indicate that experimental errors may have been made and the assay should consequently be repeated in such an event.

Although this assay has been applied to a limited number of samples, there are already some interesting trends immerging about sample quality according to tissue type. For example, it appears that maize kernels tend to contain more endogenous DNA than maize cobs, perhaps due to the protective seed coat. Further insights into DNA preservation related to environmental conditions, depositional contexts, and taphonomic processes would be invaluable for the archaeological and paleontological communities, but are not yet available given the small sample size. It is also not currently possible to state the maximum age, or more appropriately, thermal age [48], of samples which can be tested in the assay. Ultimately the DNA in an ancient plant sample will become so fragmented that none of the markers will successfully amplify. Of course, the primers which target shorter loci–plant nuclear and bacterial markers–will amplify in samples with higher amounts of DNA fragmentation. Therefore, it will still be possible to have some indication of endogenous DNA content in samples with a mean DNA fragment size <80 bp. If endogenous DNA fragments are only 60 bp, as in many charred plant remains [29], [49], the assay will fail to work. On the other hand, the assay should accommodate waterlogged samples, assuming they have sufficiently long DNA molecules, with the above caveat that cpDNA from algae may occasionally give a false signal, although the nuDNA marker can be used to confirm the presence of endogenous DNA.

DNA damage, in the form of abasic sites and strand lesions [10], is a common trait of aDNA molecules and can result in amplification failures. In terms of the assay, one could argue the endogenous DNA content would be underestimated. However, the issue may be inconsequential when the assay is used to identify which ancient samples are best suited for shotgun sequencing. For example, if two samples are being considered for shotgun sequencing to answer a given research question, the samples more likely than not came from similar contexts; accordingly, the samples would have similar levels of DNA damage and amplification inefficiencies. Therefore, even if most endogenous DNA molecules are damaged in a set of samples, the assay will still help identify the best candidate for further analyses. If all DNA in a set of samples is fragmented to the point that none of the markers will amplify, the assay cannot provide any guidance; while such samples are not necessarily are devoid of endogenous DNA, the resultant shotgun sequencing data will likely be very challenging to analyze and interpret.

Conventional genetic analyses of ancient plant samples have already provided many important insights for archaeology, paleoecology, and paleontology. Nevertheless, Palmer *et al.* [50] anticipate that high-throughput sequencing will revolutionize the field, giving researchers new tools with which to investigate more genetic markers from even older plant samples, ultimately providing keener understandings of domestication and evolution. Compared to blindly shotgun sequencing ancient plant remains, this qPCR assay provides useful insights for selecting a sample and predicting the quality of data achievable through more in depth testing. We have shown that the assay correctly identifies the top candidates, and may even help pick between high quality specimens. By prescreening ancient plant samples, researchers can prevent spending unnecessary time and resources on lower-quality samples. This is an important consideration in plant aDNA research because the number of samples available for testing frequently outweighs available funding. The simplicity and flexibility of this method allows it to be easily deployed into nearly any aDNA laboratory, as it does not require the use of expensive probes or problematical standards. Thus it can serve as an important first step to test the DNA quality of a set of ancient plant samples.

## Supporting Information

Spreadsheet S1 **Microsoft Excel workbook with worksheets for master mix setup, microwell plate layout, and automated quality-checking and analysis of qPCR results.**
(XLSX)
